# Lack of Efficacy of High-Titered Immunoglobulin in Patients with West Nile Virus Central Nervous System Disease

**DOI:** 10.3201/eid2511.190537

**Published:** 2019-11

**Authors:** John W. Gnann, Amy Agrawal, John Hart, Martha Buitrago, Paul Carson, Diane Hanfelt-Goade, Ken Tyler, Jared Spotkov, Alison Freifeld, Thomas Moore, Jorge Reyno, Henry Masur, Penelope Jester, Ilet Dale, Yufeng Li, Inmaculada Aban, Fred D. Lakeman, Richard J. Whitley, for the National Institute of Allergy and Infectious Diseases Collaborative Antiviral Study Group

**Affiliations:** University of Alabama at Birmingham, Birmingham, Alabama, USA (J.W. Gnann, Jr., P. Jester, I. Dale, Y. Li, I. Aban, F.D. Lakeman, R.J. Whitley);; National Institutes of Health Clinical Center, Bethesda, Maryland, USA (A. Agrawal, H. Masur);; University of Arkansas for Medical Sciences, Little Rock, Arkansas, USA (J. Hart);; Idaho Falls Infectious Diseases PLLC, Idaho Falls, Idaho, USA (M. Buitrago);; North Dakota State University, Fargo, North Dakota, USA (P. Carson);; University of New Mexico, Albuquerque, New Mexico, USA (D. Hanfelt-Goade);; University of Colorado at Denver Anschutz Medical Campus, Aurora, Colorado, USA (K. Tyler);; Kaiser Permanente South Bay Medical Center, Harbor City, California, USA (J. Spotkov);; University of Nebraska Medical Center, Omaha, Nebraska, USA (D. Freifeld);; Via Christi Hospital St. Francis, Wichita, Kansas, USA (T. Moore);; Infectious Diseases Consultations, Rapid City, South Dakota, USA (J. Reyno)

**Keywords:** West Nile virus, immunoglobulin, Omr-IgG-am, encephalitis, flavivirus, Polygam, viruses, WNV, central nervous system disease, neuroinvasive disease, United States, North America

## Abstract

West Nile Virus (WNV) can result in clinically severe neurologic disease. There is no treatment for WNV infection, but administration of anti-WNV polyclonal human antibody has demonstrated efficacy in animal models. We compared Omr-IgG-am, an immunoglobulin product with high titers of anti-WNV antibody, with intravenous immunoglobulin (IVIG) and normal saline to assess safety and efficacy in patients with WNV neuroinvasive disease as part of a phase I/II, randomized, double-blind, multicenter study in North America. During 2003–2006, a total of 62 hospitalized patients were randomized to receive Omr-IgG-am, standard IVIG, or normal saline (3:1:1). The primary endpoint was medication safety. Secondary endpoints were morbidity and mortality, measured using 4 standardized assessments of cognitive and functional status. The death rate in the study population was 12.9%. No significant differences were found between groups receiving Omr-IgG-am compared with IVIG or saline for either the safety or efficacy endpoints.

## Abstract 2

Immunoglobulin administered to adults with neuroinvasive disease appeared to be safe but was not demonstrated to improve clinical outcomes.

West Nile virus (WNV) is a mosquitoborne flavivirus that causes a spectrum of human illnesses, ranging from asymptomatic infection to an undifferentiated febrile syndrome (West Nile fever) and potentially lethal neuroinvasive diseases, including encephalitis and myelitis (*1*–*5*). Since its appearance in New York, USA, in 1999, WNV has become a seasonal endemic infection across North America (*5*–*7*). During 1999–2017, a total of 48,183 cases of WNV infection were reported to the Centers for Disease Control and Prevention (CDC), of which 22,999 were defined as neuroinvasive disease (*8*). Among patients with neuroinvasive disease, the mortality rate is 8%–12% (*5*,*8*,*9*). The number of reported cases of WNV disease in the United States averaged ≈2,200 cases annually during 2013–2017, although the true incidence is certainly much higher (*8*,*10*,*11*). Currently, no vaccine or drug has been approved by the Food and Drug Administration for prevention or treatment of human WNV infection.

The National Institute of Allergy and Infectious Diseases Collaborative Antiviral Study Group initiated a clinical trial of immunotherapy for patients with WNV encephalitis or myelitis using Omr-IgG-am (OMRIX Biopharmaceuticals, Tel Aviv, Israel), an immunoglobulin product that contains high titers of WNV IgG. Murine model experiments demonstrated that anti-WNV globulin administered near the time of infection was highly effective at preventing disease and death (*12*). Anecdotal cases of successful treatment of human WNV with passive immunotherapy have been reported (*13*–*16*). We conducted this phase I/II study to assess the safety and potential efficacy of Omr-IgG-am for treatment for hospitalized adults with WNV neuroinvasive disease.

## Methods

### Design

During 2003–2006, we enrolled patients into a prospective, randomized, double-blind, placebo-controlled trial of Omr-IgG-am, a human immunoglobulin preparation that had a WNV plaque-reduction neutralization titer of 1:200. We compared Omr-IgG-am with 2 controls: standard intravenous (IV) immunoglobulin (IVIG) (Polygam S/D; Baxter, https://www.baxter.com), derived from US sources and containing no detectable WNV IgG; and normal saline (NS) for IV administration. One hundred patients meeting entry criteria were to be randomized in a 3:1:1 ratio (60 for Omr-IgG-am, 20 for Polygam, and 20 for NS) in blocks of 5. Randomization was implemented with a web-based system developed and maintained by the Data Coordinating Center at the University of Alabama at Birmingham (Birmingham, AL, USA). Randomized patients received a single intravenous dose of study medication on day 1. Patients were followed for 90 days after dosing. All investigators and patients remained blinded for the duration of the study.

The 2 active dosage cohorts (0.5 g/kg and 1.0 g/kg of Omr-IgG-am) were to accrue sequentially. However, because of slow enrollment, impending expiration of Omr-IgG-am stock, and difficulty locating supplies of Polygam free of WNV IgG, the protocol was amended in 2006 to allow continued enrollment in the 0.5 g/kg cohort and to forgo the planned 1.0 g/kg cohort.

### Endpoints

The primary endpoint was safety and tolerability of the study medications at day 90 postenrollment. The safety endpoint was defined by the number of serious adverse events (SAEs), regardless of relationship to study drug. The estimated efficacy of Omr-IgG-am in reducing illness and death among patients with confirmed WNV disease (a secondary endpoint) was defined by a functional score (on day 90 after randomization) based on the results of 4 standardized assessments of cognitive and functional status: the Barthel Index (BI), the Modified Rankin Scale (MRS), the Glasgow Outcome Score (GOS), and the Modified Mini Mental State Examination (3MS) (*17*–*19*). We compared outcomes for the patients receiving Omr-IgG-am and those who received control interventions. Other secondary endpoints included the proportion of patients in each group returning to preillness baseline function as assessed by the BI and MRS, and each patient’s improvement at 3 months compared with the patient’s worst prior evaluation.

### Study Population

Participants were enrolled while hospitalized at community or academic medical centers; follow-up visits occurred at outpatient clinics. Two categories of participants were enrolled. The first included hospitalized patients >18 years of age with new-onset (<4 days’ duration) encephalitis (altered level of consciousness, dysarthria, or dysphagia), myelitis (asymmetric extremity weakness without sensory abnormality), or both. In addition, the cerebrospinal fluid (CSF) analyses (performed within the previous 96 hours) were required to show pleocytosis (>4 leukocytes/mm^3^) and negative tests for other pathogens. The second eligibility category included adults who were hospitalized without encephalitis or myelitis but who had positive WNV IgM or PCR results, as well as clinical findings compatible with WNV infection and a risk factor for the development of WNV neurologic disease (>40 years of age or immunocompromised patient >18 years of age). Confirmation of acute WNV infection by positive WNV IgM serologic results or PCR detection of WNV RNA in blood or CSF was required for inclusion in the efficacy analyses (*20*–*22*).

### Study Procedures

After verifying inclusion and exclusion criteria and obtaining informed consent, patients were randomized to 1 of the 3 treatment arms. Medical history and physical examination were recorded. Detailed neurologic examinations were conducted (BI, MRS, the GOS, and the 3MS), along with an evaluation of pre-illness functional status.

We obtained CSF samples for WNV serologic testing and PCR before starting the study and performed brain magnetic resonance imaging (MRI) studies before the study and on day 30. We examined participants on days 1–7, 14, 30, and 90. We obtained blood samples for safety laboratory studies (including complete blood count, hemoglobin A1c, platelet count, blood urea nitrogen, creatinine, creatinine phosphokinase, liver enzymes, international normalized ratio, glucose, electrolytes, amylase, and lipase); WNV, HIV, HBV, and parvovirus B19 serologic testing; and PCR for WNV, HIV, HBV, HCV, and parvovirus B19.

The unblinded research pharmacist calculated the volume of study medication. Bottles of study medication and tubing were covered with opaque plastic covers to maintain blinding. We infused study medication intravenously using a 15-µ filter at an initial rate of 0.0083 mL/kg/min, gradually increased to a maximum rate of 3 mL/min.

### Site Monitoring and Regulatory Oversight

A total of 71 sites in the United States and Canada completed regulatory requirements for enrollment (although the list of active sites varied from year to year); participants were successfully enrolled at 24 sites. The clinical trial was conducted in accordance with the ethical standards of the Helsinki Declaration. The protocol required approval by a local institutional review board (IRB) or ethics committee before enrollment could proceed; we obtained written informed consent from each participant or a legal guardian. All sites were independently monitored at selected time points and at the completion of the study. A data and safety monitoring board oversaw the study.

### Statistical Analyses

We performed statistical analyses using SAS 9.1 software (https://www.sas.com) and StaXact 4.0 (https://www.cytel.com/software/statxact) for exact statistical methods. We analyzed data using standard descriptive statistics and used Fisher’s exact test to explore associations for categorical variables between the treatment arm and the 2 control arms. Nonparametric statistical methods used a Wilcoxon test for comparison of continuous variables between the treatment arm and the 2 control arms. No interim analyses were planned.

### Sample Size Determinations

Because the study was a phase I/II safety study with a primary objective of estimating the rate of serious adverse events, we did not plan formal tests of hypotheses. Thus, we did not determine the sample size by power analysis. A total sample size of 100 participants was planned (60 Omr-IgG-am, 20 Polygam, and 20 NS). With the assumption that the true adverse event rate was no more than 30%, the 2-sided 95% CIs of the estimated SAE rates were expected to be 18.4%–41.6% for Omr-IgG-am and 9.9%–50.1% for the 2 control arms.

### Data Analyses

We included all 62 randomized participants in the safety analysis (intent-to-treat) and calculated estimates of adverse event rates with 2-sided exact 95% CIs for each treatment arm. We performed efficacy analyses on 55 study participants with confirmed WNV infection, although this phase I/II safety study was not sufficiently powered to detect small-to moderate differences in outcome among the treatment groups. The efficacy endpoint was a combination of illness and death as defined by a functional score calculated 90 days after randomization. The endpoint was based on the results of the BI, GOS, MRS, and 3MS. We placed each participant into 1 of 4 categories: dead, severely impaired, mildly or moderately impaired (but still able to function independently), and normal. We further dichotomized each category into unfavorable and favorable medical outcomes, according to predetermined cut points. Scoring in the unfavorable category on any of the 4 scales placed that participant in the unfavorable category overall.

### Conduct of the Study

The first participant was enrolled in September 2003 and the last was enrolled in September 2006. Because of slow accrual and other factors, the study was terminated in December 2006 at the recommendation of the data and safety monitoring board.

## Results

### Patient Disposition

A total of 242 patients were screened, but only 64 (26%) met the entry criteria. We did not tabulate reasons for study exclusion. Two potential participants were withdrawn before randomization. Thus, we randomized 37 patients to Omr-IgG-am, 12 to Polygam, and 13 to NS (Figure). Thirty-three of the patients in the Omr-IgG-am group, 11 in the Polygam group, and 11 in the NS group were available for follow-up at day 90. Of the 62 patients randomized, 11 terminated prematurely, 8 because of death (mortality rate 12.9%). Three (42.9%) of the 7 participants who did not have laboratory evidence of acute WNV infection died.

**Figure:**
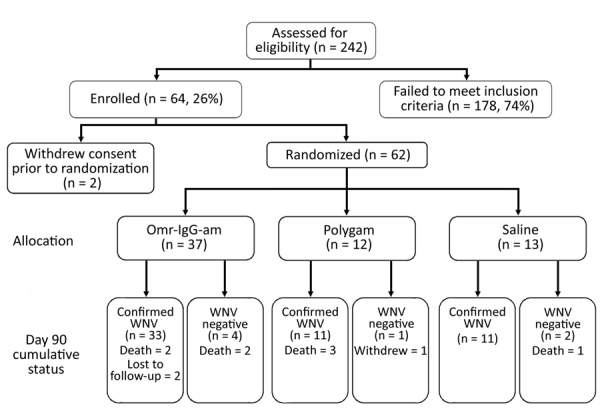
Patient enrollment, allocation, and final status in study of treatments for West Nile virus central nervous system disease.

### Study Population

Most of the patients were Caucasian (73%) and male (66%); mean age was 56 years (Table 1). We noted no baseline differences among any of the 3 randomization groups. Diagnosis of acute WNV infection was serologically confirmed in 55 patients; only 3 were positive for WNV by blood PCR. Most participants already carried a laboratory-confirmed diagnosis of WNV infection at the time of referral to the study.

**Table 1:** Demographics and clinical characteristics of patients in study of treatments for West Nile virus central nervous system disease, by treatment arm*

Characteristic	Omr-IgG-am, n = 37	Polygam, n = 12	Normal saline, n = 13	Total, n = 62
Race				
Caucasian/not Hispanic	24 (64.9)	10 (83.3)	11 (84.6)	45 (72.6)
Black/not Hispanic	2 (5.4)	0	1 (7.7)	3 (4.8)
Hispanic	8 (21.6)	1 (8.3)	1 (7.7)	10 (16.1)
Other	3 (8.1)	1 (8.3)	0 (0)	4 (6.5)
Sex				
M	25 (67.6)	7 (58.3)	9 (69.2)	41 (66.1)
F	12 (32.4)	5 (41.7)	4 (30.8)	21 (33.9)
Age, y, mean ± SE	56.2 ± 2.2	54.0 ± 4.1	58.4 ± 4.8	56.2 ± 1.8
Cerebrospinal fluid				
Leukocytes, cells/µL, mean ± SE (median)	207.7 ± 40.3 (124)	187.4 ± 105.5 (58)	146.6 ± 85.8 (37)	192.3 ± 35.3 (95.5)
% Lymphocytes, mean ± SE (median)	53.9 ± 5.0 (52.5)	39.3 ± 10.0 (36.5)	50.2 ± 11.6 (34)	50.1 ± 4.3 (43)
Time from admission to drug infusion, d, mean ± SE (median)	2.9 ± 0.5 (2)	2.8 ± 0.5 (2.5)	2.0 ± 0.4 (2)	2.7 ± 0.3 (2)
Disease group and risk factors				
With encephalitis/myelitis	25 (64.7)	9 (75.0)	11 (84.6)	44 (71.0)
Without encephalitis/myelitis	12 (32.4)	3 (25.0)	2 (15.4)	17 (27.4)
Hematologic malignancy	1 (8.3)	0	0	1 (5.9)
Diabetes mellitus	2 (16.7)	0	0	2 (11.8)
Bone marrow transplant	1 (8.3)	0	0	1 (5.9)
Immunosuppressive medications	1 (8.3)	0	0	1 (5.9)

*Values are no. (%) patients except as indicated. In comparing Omr-IgG-am with Polygam and Omr-IgG-am with normal saline, no significant differences were identified for characteristics listed in this table (all p values >0.05). Denominators vary according to the number of evaluable patients or assessments.

### Clinical Characteristics

Because we found no differences among any of the 3 randomization groups, we summarized the clinical characterization for all 55 patients with confirmed WNV infection. The most common symptoms were fever >38°C (80%), chills/rigors (75%), headache (78%), nuchal rigidity (47%), photophobia (33%), myalgia (78%), arthralgia (33%), nausea (76%), vomiting (60%), diarrhea (46%), shortness of breath (24%), and rash (27%). Detailed descriptions of the neurologic findings in this study cohort were published previously (*23*). Of the 44 participants with abnormal CSF findings at initial evaluation, median values were protein 90 mg/dL, glucose 62 mg/dL, erythrocytes 10 cells/mm^3^, and leukocytes 96 cells/mm^3^ (lymphocytes 62%, neutrophils 38%).

### Safety and Tolerability

As expected for this population of seriously ill patients, large numbers of adverse events (AEs) were recorded (Table 2). A total of 738 AEs were reported for 58 participants (12.72 AEs/person). The most commonly reported treatment-related AE was hypertension occurring during infusion of the test drug (Table 3). Two grade 3–4 laboratory toxicities (both decreased hematocrit) were reported, both occurring in Omr-IgG-am recipients.

**Table 2:** Summary of AEs in intent-to-treat population of patients in study of treatments for West Nile virus central nervous system disease, by treatment arm*

Characteristic	Omr-IgG-am, n = 37	Polygam, n = 12	Normal saline, n = 13	Total, n = 62
AEs	514	106	118	738
patients with an AE	36 (97.3)	11 (91.7)	58 (93.5)	58 (93.5)
AEs per patient	14.28	9.64	10.73	12.72
Relationship to treatment†				
Unrelated	482 (93.8)	99 (93.4)	113 (95.8)	694 (94)
Related	29 (5.6)	7 (6.6)	5 (4.2)	41 (5.6)
Not stated	3 (0.6)	0	0	3 (0.4)
Severity of AE†				
Mild	226 (44.0)	45 (42.5)	61 (51.7)	332 (45.0)
Moderate	221 (43.0)	34 (32.1)	45 (38.1)	300 (40.7)
Severe	54 (10.5)	13 (12.3)	9 (7.6)	76 (10.3)
Life-threatening	13 (2.5)	14 (13.2)	3 (2.5)	30 (4.1)

*Values are no. (%) except as indicated. In comparing Omr-IgG-am with Polygam and Omr-IgG-am with normal saline, no significant differences were identified for characteristics listed in this table (all p values >0.05) except for mild severity Omr-IgG-am vs. Polygam (p<0.01). AE, adverse event. †As assessed by the investigator.

**Table 3:** Most commonly reported treatment-related adverse events in intent-to-treat population in study of treatments for West Nile virus central nervous system disease, by treatment arm*

Adverse event	Omr-IgG-am, n = 37	Polygam, n = 12	Normal saline, n = 13	Total, n = 62
Hypertension	7	0	2	9
Dosing error	6	0	2	8
Elevated transaminases	3	1	0	4
Fever, chills	3	1	0	4
Shortness of breath	0	3	0	3
Rash, pruritus	2	0	0	2
Chest pain	2	0	0	2
Other	6	2	1	9
Total	29	7	5	41

*Values are no. patients.

Safety was defined by the total number of SAEs among the 62 randomized participants, regardless of relatedness to study drug administration. Overall, 29 participants (46.8%) experienced 63 SAEs (29 with Omr-IgG-am, 25 with Polygam, 9 with NS; Table 4). The estimated SAE rates (with 2-sided 95% confidence intervals) were 51.4% (range 35.3%–67.7%) for recipients of Omr-IgG-am, 58.3% (range 30.4%–86.2%) for recipients of Polygam, and 23.1% (range 0.2%–46.0%) for those who received NS. The differences in frequency of SAEs among the 3 treatment groups were not statistically significant. A larger number of neurologic SAEs were reported in the Polygam group (Table 5), although the types of events were highly divergent (declining mental status, quadriparesis, cranial nerve palsies, tremor, seizures) and likely attributable to WNV neuroinvasive disease.

**Table 4:** Summary of SAEs in intent-to-treat population in study of treatments for West Nile virus central nervous system disease, by treatment arm*

Characteristic	Omr-IgG-am, n = 37	Polygam, n = 12	Normal saline, n = 13	Total, n = 62
SAEs	29	25	9	63
Patients with an SAE	19 (51.4)	7 (58.3)	3 (23.1)	29 (46.8)
SAEs per patient	1.53	3.57	3.0	2.17
Relationship to treatment†				
Unrelated	27 (93.1)	24 (96.0)	7 (77.8)	58 (92.1)
Related	2 (6.9)	1 (4.0)	2 (22.2)	5 (7.9)
Severity†				
Mild	1 (3.4)	0	0	1 (1.6)
Moderate	4 (13.8)	3 (12.0)	0	7 (11.1)
Severe	9 (31.0)	8 (32.0)	4 (44.4)	21 (33.3)
Life-threatening	11 (37.9)	11 (44.0)	4 (44.4)	26 (41.3)
Death	4 (13.8)	3 (12.0)	1 (11.1)	8 (12.7)

*Values are no. (%) except as indicated. In comparing Omr-IgG-am with Polygam and Omr-IgG-am with normal saline, no significant differences were identified for characteristics listed in this table (all p values >0.05) except for mild severity Omr-IgG-am vs. Polygam (p<0.01). SAE, serious adverse event. †As assessed by the investigator.

**Table 5:** Most commonly reported serious adverse events in intent-to-treat population in study of treatments for West Nile virus central nervous system disease, by treatment arm*

Serious adverse event	Omr-IgG-am, n = 37	Polygam, n = 12	Normal saline, n = 13	Total, n = 62
Respiratory failure	8	7	2	17
Neurologic event or mental status decline	1	11	1	13
Cardiac event	4	1	0	5
Anemia	2	1	0	3
Leukopenia	1	0	2	3
Urinary tract infection	1	0	1	2
Pneumonia	2	0	0	2
Pulmonary embolism	1	0	1	2
Atelectasis	0	2	0	2
Pleural effusion	0	1	1	2
Other	9	2	1	12
Total	29	25	9	63
*Values are no. patients.				

Five SAEs were assessed by the investigator to be possibly, probably, or definitely related to the study medication (Table 4). Two events (chest pain and leukopenia, both assessed as possibly) occurred in Omr-IgG-am recipients; both resolved. One SAE (respiratory distress, assessed as probably) occurred in a Polygam recipient and resolved. Two instances of neutropenia (both assessed as possibly) were reported in NS recipients and resolved.

To monitor the possibility of transmission of other viral pathogens by the immunoglobulin preparations, participants were screened preinfusion and on day 30. No participant was positive for HBV or HIV. Two patients (1 recipient of Omr-IgG-am, 1 recipient of Polygam) had negative parvovirus B19 IgG titers preinfusion but had detectable antibodies at follow-up; both of these patients had negative parvovirus IgM titers and PCR assays. This finding likely represents antibody passively acquired from the immunoglobulin infusion rather than acute parvovirus infection. One patient tested positive for hepatitis C virus by both serologic testing and PCR preinfusion and remained positive on day 90.

### Efficacy

For each test instrument (BI, 3MS, GOS, and MRS), composite scores measured at the time of enrollment indicated impaired neuropsychological function, which improved over 90 days of follow-up, consistent with the natural history of resolving WNV neurologic disease (Table 6). No significant differences in outcomes were apparent for the 3 treatment groups; therefore, summary statistics allow assessment of day 90 outcomes for the combined population. By 3MS, 50.9% of patients were determined to be normal/unimpaired, 18.2% had mild or moderate impairment, 12.7% were severely impaired, and 9.1% died. When the BI, GOS, and MRS tests were applied to the same population, the percentage of patients who were evaluated as normal/unimpaired were 47.3% by BI, 36.4% by GOS, and 14.5% by MRS; the proportion classified as severely impaired was 27%–29% by each of these 3 instruments.

**Table 6:** Summary of impairment and death at day 90 after randomization for patients with confirmed West Nile virus in study of treatments for West Nile virus central nervous system disease, by treatment arm*

Instrument	Omr-IgG-am, n = 33	Polygam, n = 11	Normal saline, n = 11	Total, n = 55
Modified Mini-Mental Status Examination				
Normal, score >88	14 (42.4)	6 (54.5)	8 (72.7)	28 (50.9)
Mild/moderately impaired but independent, score 78–88	8 (24.2)	0	2 (18.2)	10 (18.2)
Severely impaired, score <78	5 (15.2)	1 (9.1)	1 (9.1)	7 (12.7)
Dead, score 0	2 (6.0)	3 (27.3)	0	5 (9.1)
Not done/lost to follow-up	4 (12.1)	1 (9.0)	0	5 (9.1)
Barthel Index				
Normal, score >94	15 (45.5)	5 (45.5)	6 (54.5)	26 (47.3)
Mild/moderately impaired but independent, score 90–94	1 (3.0)	0	2 (18.2)	3 (5.5)
Severely impaired, score <90	11 (33.3)	2 (18.2)	3 (27.3)	16 (29.1)
Dead, score 0	2 (6.1)	3 (27.3)	0	5 (9.1)
Not done/lost to follow-up	4 (12.1)	1 (9.0)	0	5 (9.1)
Glasgow Outcome Score				
Normal, score 5	10 (30.3)	3 (27.3)	7 (63.6)	20 (36.4)
Mild/moderately impaired but independent, score 4	9 (27.2)	2 (18.2)	1 (9.1)	12 (21.8)
Severely impaired, score 2–3	9 (27.2)	3 (27.3)	3 (27.3)	15 (27.3)
Dead, score 1	2 (6.0)	3 (27.3)	0	5 (9.1)
Not done/lost to follow-up	3 (9.0)	0	0	3 (5.5)
Modified Rankin Scale				
Normal, score 0	4 (12.1)	0	4 (36.4)	8 (14.5)
Mild/moderately impaired but independent, score 1–3	15 (45.5)	5 (45.5)	4 (36.4)	24 (43.6)
Severely impaired, score 4–5	10 (30.3)	3 (27.3)	3 (27.3)	16 (29.1)
Dead, score 6	2 (6.0)	3 (27.3)	0	5 (9.1)
Not done	2 (6.0)	0	0	2 (3.6)

*Values are no. (%) patients. In comparing Omr-IgG-am with Polygam and Omr-IgG-am with normal saline, we found no significant differences with respect to the impairment and death categories (excluding “not done”) for all instruments listed on this table (p > 0.05 for all values).

We further dichotomized outcomes into favorable and unfavorable (Table 7). We found no significant differences in the proportion of patients experiencing an unfavorable outcome at day 90 between treatment and control (although there was again a nonsignificant trend toward better outcomes in the NS group). Overall, 51% of patients had a favorable outcome. We determined the proportion of patients returning to preillness baseline at day 90 for each randomization group. By the BI and the MRS, the 2 most sensitive indices, 45.9% and 32.8% of patients returned to their preillness status, respectively.

**Table 7:** Summary of unfavorable outcomes at day 90 after randomization of patients with confirmed West Nile virus in study of treatments for West Nile virus central nervous system disease*

Regimen	No. (%) patients			Odds ratio (95% CI)
	Favorable	Unfavorable	Missing	
Omr-IgG-am, n = 33	15 (45.5)	17 (51.5)	1 (3.0)	Referent
Polygam, n = 11	5 (45.5)	6 (54.5)	0	1.012 (0.198–4.975)
Normal saline, n = 11	8 (72.7)	3 (27.3)	0	3.238 (0.606–21.959)
Total confirmed, n = 55	28 (50.9)	26 (47.2)	1 (1.8)	


*Favorable/unfavorable determinations made on the basis of results of 4 standardized assessments of cognitive and functional status: Barthel Index (favorable >90, unfavorable <90), Modified Rankin Scale (favorable 0–3, unfavorable 4–6), Glasgow Outcome Score (favorable 4 or 5, unfavorable <4), and the Modified Mini Mental State Examination (favorable >78, unfavorable <78).

The median duration of hospital stay was 10 days for the Omr-IgG-am group, 12 days for the Polygam group, and 8.5 days for the NS group. Of the 62 patients enrolled in the study, 23 (37%) required intensive care unit (ICU) management. We found no differences in the duration of ICU stay (median 13 days) among the 3 treatment groups. Six patients required mechanical ventilation (median duration 5 days).

### Virologic Studies

All 55 patients initially had positive WNV IgM serologic test results; 36 (67.9%) of 53 patients were WNV IgG positive preinfusion. Reverse transcription PCR for WNV RNA in blood was positive for only 3 (5.9%) of 51 patients before infusion of study medication; no patient had a positive WNV PCR result from blood on day 3. Of the 49 patients for whom day 90 serologic data were available, 40 (81.6%) were persistently positive for WNV IgM and 47 (95.9%) for WNV IgG.

## Discussion

Because preliminary data from animal models and case reports suggested that immunotherapy could alter the outcome of WNV neurologic infection, the National Institute of Allergy and Infectious Diseases Collaborative Antiviral Study Group initiated a clinical study to determine the safety and potential efficacy of a high-titered immunoglobulin product in patients with WNV neuroinvasive disease. The trial was terminated prematurely because of slow accrual and reduced availability of study products. At the time of study termination in 2006, the Polygam supply derived from US sources contained measurable titers of WNV IgG and was no longer an acceptable control.

Looking at recorded SAEs, deaths, and laboratory parameters, we found no differences in safety and tolerability among Omr-IgG-am (0.5 g/kg), Polygam, and NS. Illness outcomes, measured by a panel of 4 neuropsychological test instruments, were not statistically different among the 3 groups. Although the results did not meet statistical significance (in part because of the small sample size), we found a persistent trend toward better outcomes (both illness and death) in the NS group compared with the immunoglobulin groups (Tables 6, 7). Although the validity of this observation is unconfirmed, we do not recommend the administration of immunoglobulin products to patients with neuroinvasive WNV disease until further research can be conducted to establish the relative risk–benefit profile.

The study protocol was designed to capture patients as early as possible in their clinical courses, when immunotherapy was most likely to be beneficial. Unfortunately, patients often entered the study pool later, after a diagnosis of WNV infection had been confirmed by laboratory testing. Delays in enrollment and study drug administration could have diminished the potential efficacy of Omr-IgG-am. By the time symptomatic neurologic disease was present, the infection had probably progressed to a point at which the administration of passive immunotherapy was unlikely to be beneficial.

In this study population of relatively healthy middle-aged persons, 15% (as assessed by MRS) to 50% (as assessed by BI) returned to baseline function. Only 33%–46% of patients returned to their preillness state (as defined retrospectively by a family member). These data differ somewhat from proportions of patients experiencing normal or mild to moderate impairment reported by other investigators (*24*–*28*). Other published WNV case series had different demographic and disease characteristics and used different definitions, making interstudy comparisons problematic. Our population had a relatively high percentage of patients requiring ICU care and extended durations of hospitalization.

Effective therapy for WNV neuroinvasive disease remains an unmet medical need. A human monoclonal antibody directed against WNV has shown activity in animal models (*29*,*30*) but remains unproven for human infection (*31*,*32*). Antiviral drugs that can be initiated early in the course of WNV disease are urgently needed (*33*–*35*).

We learned several lessons that will inform the design of future studies of therapies for WNV disease. At various time points, this clinical trial activated 71 individual sites in 28 US states and 3 Canada provinces but was still unable to achieve full enrollment. The challenges encountered during the conduct of the study were numerous. First, and most notably, the precise geographic localization of emerging vectorborne illnesses is difficult to predict. WNV infection occurs seasonally (usually July–October in North America) and in scattered geographic locations. We worked closely with our collaborators at CDC but were unable to project with sufficient precision where the incidence of WNV disease would be highest in the subsequent season. Even when we correctly predicted geographic regions where disease activity was high, it was extremely difficult to activate sufficient study sites quickly. Furthermore, WNV infection is predominantly a rural disease, whereas many of our study sites were located in urban areas. Second, it was often difficult to refer potential participants to active study sites. Investigators received numerous calls regarding WNV patients who were hospitalized at nonparticipating medical centers, some even in the same city. However, logistical and financial constraints prevented most of these patients from being transferred to a site with an IRB-approved protocol in place. Third, most patients were considered for enrollment in the study only after WNV infection had been confirmed. The study was designed to enable enrollment of suspected WNV patients (before laboratory confirmation) to expedite early therapy, but this rarely occurred, as demonstrated by the mean time from admission to study drug administration (2.6 days). Animal model data have indicated that passive immunotherapy of WNV infection with exogenous antibodies is most effective if instituted very early in the course of infection.

Finally, there were regulatory constraints, as we have described previously (*36*). The median time required to obtain IRB approval at US medical centers was ≈6 months (*36*). Consequently, many potential participants could not be enrolled because sites failed to receive IRB approval and activate the protocol in a timely manner. The availability of a central IRB could have shortened site registration time considerably and potentially enhanced patient enrollment. As a result of unpredictable geographic variation, fluctuating incidence, and seasonal enrollment windows, an agile and flexible universal IRB system will be mandatory if future large-scale clinical trials of therapies for emerging vectorborne infectious diseases (e.g., WNV, Chikungunya, dengue, Zika virus) are to be successfully performed in the United States.

Appendix List of investigators and coordinators of study of patients with West Nile virus central nervous system disease.
