# Supplementary material for: Lack of Efficacy of High-Titered Immunoglobulin in Patients with West Nile Virus Central Nervous System Disease
Source: Emerg Infect Dis. 2019 Nov;25(11):2064–73. doi: 10.3201/eid2511.190537 (PMC6810207; doi:10.3201/eid2511.190537)
Supplement: Appendix — List of investigators and coordinators of study of patients with West Nile virus central nervous system disease. [file 19-0537-Techapp-s1.pdf]

Article DOI: https://doi.org/10.3201/eid2511.190537

Lack of Efficacy of High-Titered Immunoglobulin in Patients with West Nile Virus Central Nervous System Disease

Appendix

The authors acknowledge and thank the investigators and coordinators listed in the following table, who enrolled participants in this study.

Investigator	Affiliation	Study coordinator
Dr. Amy Guillet Agrawal	National Institutes of Health Clinical Center, Bethesda, Maryland (now at Holy Cross Hospital, Silver Spring, MD)	Deba Reda, RN
Dr. Fred Aoki	University of Manitoba Health Sciences Center, Winnipeg, Manitoba, Canada	Elizabeth Elsie Friesen, RN
Dr. Aristides Assimacopoulos	Avera Research Institute, Sioux Falls, South Dakota (now at St. Alexius Medical Center, Hoffman Estates, IL)	Julie Fieldsend, RN
Dr. Martha Buitrago	Idaho Falls Infectious Diseases, Idaho Falls, Idaho	Shannon Neff, RN
Dr. Paul Carson	Merit Healthcare System, Fargo, North Dakota (now at North Dakota State University, Fargo, ND)	Brandee Godfrey, BA
Dr. Mazen DiMachkie	University of Texas at Houston, Houston, Texas (now at University of Kansas Medical Center, Kansas City, KS)	Sandi Shaw, RN
Dr. Philip Fracica	St. Joseph's Hospital and Medical Center, Phoenix, Arizona (now at Bothwell Regional Health Care, Sedalia, MO)	Kelli Williamson, RN, MPH
Dr. Alison Freifeld	University of Nebraska Medical Center, Omaha, Nebraska	Penny Hardiman, RN,
Dr. John Gnann	University of Alabama at Birmingham, Birmingham, AL (now at Medical University of South Carolina, Charleston, SC)	Nancy Grady, RN
Dr. Diane Hanfelt-Goade	University of New Mexico, Albuquerque, New Mexico (now at Tauranga Hospital, Tauranga, New Zealand)	Julianna Ferreira
Dr. Gary Green	Kaiser Permanente Santa Rosa Medical Center, Santa Rosa, California	Susan Kirk, RN
Dr. Robert Grimm (deceased)	Good Samaritan-Legacy, Portland, Oregon	Lee McCafferty, RN
Dr. John Hagan III	St. Alexis Medical Center, Bismarck, North Dakota	Julie Wetzstein, CCRC
Dr. Daniel Hanley	Johns Hopkins Medical Institutions, Baltimore, Maryland	Susan M. Rice, RN
Dr. John Hart	University of Arkansas for Medical Sciences, Little Rock, Arkansas (now at University *of Texas* Southwestern Medical Center, Dallas, TX)	
Dr. Rodrigo Hasburn	Tulane University Health Science Center, New Orleans, Louisiana (now at Memorial Hermann Texas Medical Center, Houston, TX)	Karen Craig, RN
Dr. Daniel Hinthorn	University of Kansas Hospital, Kansas City, Kansas	Stacy McCrea-Robertson, MS
Dr. Robert S. Jones	The Reading Hospital and Medical Center, Reading, Pennsylvania	Carolyn Foster, RN, CCRC
Dr. Fred D. Lakeman	University of Alabama at Birmingham, Birmingham, Alabama (retired)	
Dr. Robert A. Larsen	Los Angeles County/University of Southern California Medical Center, Los Angeles, California (retired)	Edward Lanzo, RN
Dr. Miguel Mogyoros	Exempla St. Joseph Hospital, Denver, Colorado	Nancy Weber, RN
Dr. Thomas Moore	Via Christi Hospital St. Francis, Wichita, Kansas (now at Infectious Diseases Consultants, Wichita, KS)	Karen Wiant, RN
Dr. Richard Pollard	University of California, Davis Medical Center, Sacramento, California	Abby Olusanya, MSN, FNP, CCRP
Dr. Jorge Reyno	Infectious Disease Consultations, Rapid City, South Dakota (now at Martin Luther King, Jr. Community Hospital, Los Angeles, CA)	Rod Marchlando, RPh, PharmD
Dr. George Risi	Montana Infectious Disease Specialists, Missoula, Montana (now at US Department of Health and Human Services Biomedical Advanced Research and Development Authority, Washington, DC)	Kelly Dudley, RN
Dr. Jared Spotkov	Kaiser Permanente South Bay Medical Center, Harbor City, California	Christine H. Suh, RN

Investigator	Affiliation	Study coordinator
Dr. Kenneth L. Tyler	University of Colorado at Denver Anschutz Medical Campus, Aurora, Colorado	Graham Ray, NP
Dr. Richard J. Whitley	University of Alabama at Birmingham, Birmingham, Alabama	
